# Transactions of the New York State Medical Society for 1854

**Published:** 1854-05

**Authors:** 


*Transactions of the New York State Medical Society for* 1854. *Published by the Legislature.*

We cannot accord to this volume as much merit as has characterized some of its predecessors. The papers, though some of them valuable, are not of very general interest, and the book is made up, in part, of reports or addresses delivered before county societies, which do not, in reality, constitute any part of the transactions of the State Society.

If the necessity of making up a good-sized volume was the motive of these selections, we could understand it, but that necessity did not exist. The paper on Croup forwarded by Drs. Parker and Cock, of New York, through the hands of Dr. Alonzo Clark, has only a mention in the minutes, while it really constituted the most interesting of all the documents presented, elicited the most discussion, and possessed at least as much intrinsic value as any part of the proceedings. Dr. Van Dyck also was requested to draw up in manuscript his fine extempore account of medicine in Syria, but that, too, is forgotten.

The session itself was most interesting and profitable to the large number who were present. It is to be regretted that its printed records do not, in some measure, reflect that interest.

The Transactions open with the Annual Address delivered before the society and legislature by the president, Jenks S. Sprague, M. D.

This is a paper suited to the occasion and to the audience which listened. Commencing with a tribute to the glorious history of medicine, and to the noble character of its earlier luminaries, it soon leaves the field of the usual generalities, and goes into an extended and able argument upon the "Bill for the Promotion of Medical Science," then under the consideration of the legislature who formed a part of his audience.

It is unnecessary to recount here the train of argument employed by Dr. Sprague. It is a familiar subject to the profession, and every reader can supply them from his own heart, but the time, the place, and the audience, al l tended to make their really forcible exposition more than usually impressive.

The right so earnestly entreated is at last conceded to us. The Dissection Bill is a law—imperfect and too narrow in its restrictions—but destined to be, even in its present form, a great benefit to science, while it is not too much to hope that it may, from time to time, receive those amendments which are so evidently necessary.

The second article is an account by Dr. Wm. H. H. Parkhurst, of an extra-uterine conception, in which it is satisfactorily proved that the fœtus remained in the abdominal cavity for fifty years. The specimen obtained at the post-mortem, was exhibited to the society.

Dr. Alden March's paper on penetrating wounds of the abdomen and larynx follows. Cases of each are given, as illustrative of the points which should govern the treatment. The article is instructive, and its views, if they lack the merit of novelty are at least sound. He urges the necessity of entire rest of the intestine in wounds of the abdomen, and considers morphine as by far the best antiphlogistic to be exhibited. In wounds of the larynx he repeats the cautions of many authors against closing the wound entirely.

Article IV, by Samuel Shumway, M. D., is a brief report of a case of reduction of a dislocation of the hip joint, by what we shall persist in calling "*Reid's method.*"

It is true that Dr. Shumway has known this method since the winter of 1815–16, where, he says, he saw Dr. Nathan Smith demonstrate it upon the skeleton at the medical department of Yale College. We are willing to admit Dr. Smith's claim to this discovery, but for the score or two of gentlemen who have, since the publication of Dr. Reid's article on the subject in this Journal, risen up to proclaim that they had been familiar with the operation this forty years, we can only repeat what we have often thought—they should be presented with a large-sized leather medal in reward for the prodigious care they have taken to keep their knowledge to themselves.

Article V. is transferred from the Albany County Society's Transactions. It is the annual address delivered by J. V. P. Quackenbush, M. D., on prolapsus of the bladder. It is a good and practical paper on the subject, and if not strictly a part of the State Society's Trausactions, it is a very readable document.

It closes with a biographical sketch of the distinguished Dr. Lewis C. Beck, and also of Dr. David Martin. Such tributes to departed excellence are always interesting to the great body of the profession, and the death of such a man as Dr. Beck should not pass without a place on the records of that state society which he served so long, so faithfully, and so well.

Dr. Kneeland's address to the Onondaga County Society follows. It has the merit of being a well-written and eloquent exposition of the claims of legitimate medicine, and of some sensible recommendations as to the best means of giving interest and and efficiency to our county societies.

But to it, and much more strongly to the paper by Dr. Cash, following it, we would raise this objection. They do not in any legitimate sense tend to advance the profession. The public do not read these appeals, or if they do, they regard them as *ex-parte* statements. The profession must, by this time, be tolerably well convinced of its own dignity and honor, and we would much rather see the space occupied by these expositions, however eloquent or truthful, devoted to papers of practical interest, such as we have mentioned as omitted in this volume of transactions.

The proceedings of the society occupy the remaining pages. Among them Prof. Draper's letter to the State Censors will attract notice, and we fancy, no small amount of criticism; but we have already "said our say" upon that matter.

We are indebted to Senator Putnam for our copy of the Transactions.

